# Gene hunting in autoinflammation

**DOI:** 10.1186/2045-7022-3-32

**Published:** 2013-09-26

**Authors:** Ariane Standing, Ebun Omoyinmi, Paul Brogan

**Affiliations:** 1 Institute of Child Health, UCL, 30 Guilford Street, London WC1N 1EH, UK

**Keywords:** Genetic mapping, Autoinflammatory diseases, CAPS, NGS, CANDLE

## Abstract

Steady progress in our understanding of the genetic basis of autoinflammatory diseases has been made over the past 16 years. Since the discovery of the familial **Me**diterranean **f**e**v**er gene *MEFV* (also known as marenostrin) in 1997, 18 other genes responsible for monogenic autoinflammatory diseases have been identified to date. The discovery of these genes was made through the utilisation of many genetic mapping techniques, including next generation sequencing platforms. This review article clearly describes the gene hunting approaches, methods of data analysis and the technological platforms used, which has relevance to all those working within the field of gene discovery for Mendelian disorders.

## Introduction

The concept of autoinflammatory diseases was first proposed by McDermott and colleagues 15 years ago upon the elucidation of the genetic cause of TNF Receptor Associated Periodic Syndrome (TRAPS), the second of the prototypic periodic fevers; following just two years after the identification of *MEFV* the Familial Mediterranean Fever (FMF) gene. They used the term 'autoinflammatory’ to distinguish the fact that autoantibodies were conspicuously absent and the innate immune response seemed to predominate in the pathogenesis [1]. There are two factors which indicate autoinflammatory disease: abnormally increased inflammation, mediated predominantly by the cells and molecules of the innate immune system, and significant host predisposition (monogenic or polygenic) [2].

Infevers, an online database of known mutations [3] currently recognises 19 monogenic autoinflammatory diseases where genes have been identified and mutations are curated. Currently in the UK, six genes can be screened routinely (*MEFV*, *NLRP3* and *12*, *MVK*, *TNFRSF1A*, and *NOD2*); others are sent to reference and research labs around the world. However, as many as 60% of patients with autoinflammatory diseases do not fit with the known syndromes and/or screening for known genes is negative [2]. New syndromes are still being reported. In the last two years there has been the identification of genes for six monogenic autoinflammatory diseases. In addition, certain polygenic diseases previously considered as autoimmune are now being considered for reclassification as autoinflammatory, for example Behçet’s disease, systemic juvenile idiopathic arthritis (sJIA), ankylosing spondylitis, Crohn’s disease, psoriasis and more [4].

With the exception of the cryopyrinopathies, where IL-1 blockade has revolutionised treatment, and FMF where colchicine is a safe and effective treatment for most, there is no ideal consensus treatment for many emerging new autoinflammatory diseases. As the aetiopathogenesis remains unknown for many of these conditions, treatment relies on non-specific immunosuppression with corticosteroids and/or other empiric trials of immunosuppressants including biologics, sometimes with significant side effects and cost. In extremely severe conditions allogeneic stem cell transplantation may be offered, but this is risky, and if pathogenesis is unknown there is no guarantee that it will be effective particularly for conditions associated with mutations not restricted to the haematopoietic system. Undoubtedly, identifying causative genes and pathways will lead to better targeted treatment. It will also improve diagnostic screening for other affected family members or unrelated patients with unclassified autoinflammatory diseases. Additionally, identification of novel mutations causing autoinflammation will enable genetic counselling for the family. Thus searching for novel genetic causes of autoinflammatory disease is both beneficial to patients, as well as advancing our knowledge of these diseases.

## Review

The decision of which genes to screen for mutations can be based on: the function of the gene (candidate gene approach), location in the genome following mapping (linkage analysis and/or homozygosity mapping), or a combination of both. New next-generation sequencing technologies now permit the screening of every gene at once by sequencing whole exomes and even whole genomes. The history of the discovery of the 19 known monogenic causes of autoinflammation provides examples of the various strategies for the discovery of monogenic autoinflammatory diseases, as well as telling the story of the continued evolution in genetic techniques. Genes identified thus far, and the methods used for their discovery are summarised in Table 1. Gene discovery in polygenic disease is more complex, as each genetic variant confers only a small increase in susceptibility. Whilst this review predominantly focuses on monogenic disease, association studies in polygenic disease will also be touched upon.

**Table 1:** Summary of the genes identified to be mutated in monogenic autoinflammatory diseases, the year they were first recognised and mapping techniques which contributed to this

Year	**Disease**	**Gene**	**Methods**
1997	Familial Mediterranean Fever (FMF)	*MEFV*	Multipoint and two point linkage with RFLPs and microsatellites
			Positional cloning [5,6].
1999	TNF-Receptor Associated Periodic Syndrome (TRAPS)	*TNFRSF1A*	Multipoint linkage, two point linkage [1]. Multipoint and two-point linkage and haplotype analysis [7].
1999	Hyper IgD Syndrome (HIDS)	*MVK*	Pairwise Linkage with microsatellites [8].
2001	Cryopyrin Associated Periodic Syndromes (CAPS)	*NLRP3 (CIAS1)*	Microsatellite multipoint and two-point linkage, and haplotype analysis in MWS [9] and FCAS [10]; candidate gene CINCA [11].
2001	Blau Syndrome	*NOD2*	Microsatellites, multi and two-point linkage and haplotype analysis [12]. Candidate gene [13].
2001	Cherubism	*SH3BP2*	Microsatellite pairwise and multipoint linkage, haplotype analysis [14-16]. Candidate gene [14].
2002	Pyogenic sterile Arthritis, Pyoderma gangrenosum and Acne (PAPA)	*PSTPIP1*	Microsatellite haplotype analysis and candidate gene [17-19].
2004	Early Onset Sarcoidosis (EOS)	*NOD2*	Candidate gene [20,21].
2005	Majeed Syndrome	*LPIN2*	Microsatellites, homozygosity mapping, two and multipoint linkage and haplotype analysis [22].
2006	Recurrent Hydatidiform Mole 1 (RHM1)	*NLRP7*	Microsatellites, multipoint linkage and haplotype analysis. Candidate gene [23,24].
2008	Familial Cold Autoinflammatory Syndrome 2 (FCAS2)	*NLRP12*	Candidate gene [25].
2009	Deficiency of IL-1 Receptor Antagonist (DIRA)	*IL1RN*	Candidate gene [26].
2009	Severe infantile inflammatory bowel disease	*IL10RA, IL10RB, IL10*	Haplotype analysis and multipoint linkage with microsatellites and SNP arrays [27]. Candidate gene [28].
2011	CANDLE/JMP/NNS	*PSMB8*	SNP homozygosity mapping, parametric multipoint linkage and [29-32] Exome analysis [31,32].
2011	Deficiency of IL36 Receptor Antagonist (DITRA)	*IL36RN*	SNP array based homozygosity mapping then multipoint linkage and haplotype analysis with microsatellites [33]. Exome sequencing [34].
2012	Autoinflammation & PLCγ2-associated antibody deficiency & immune dysregulation (APLAID)	*PLCG2*	Exome sequencing [35].
2012	HOIL1 Deficiency	*RBCK1 (HOIL1)*	SNP based deletion screening and exome sequencing [36].
2013	Pustular psoriasis/ pityriasis rubra pilaris	*CARD14*	SNP multipoint linkage, microsatellite and RFLP haplotype analysis, targeted exome and candidate gene sequencing [37]. Exome and targeted-capture sequencing [38].

### Candidate gene approach

Sometimes a whole genome candidate gene approach is taken without prior mapping. *NLRP12* was identified from a whole genome candidate gene approach; Jeru and colleagues decided to screen *NLRP* genes in periodic fever patients, as NLRP proteins are involved in the recognition of microbial molecules and the activation of immune responses [25]. Similarly, after observing a positive response to anakinra (the IL-1 receptor antagonist) in a family with a neonatal onset pyogenic disorder, Aksentijevich and colleagues decided to screen genes in the IL-1 pathway and found mutations in *IL1RN* in 6 families [26]. Sometimes a candidate gene approach is applied to a smaller region after genetic mapping, see below. What is evident from these examples is that insight into the innate immune system and the pathogenesis of known inflammatory disease is useful to inform candidate gene identification in novel diseases.

### Genetic mapping

In human genetics, linkage analysis has traditionally been conducted as the first step in mapping a trait. The majority of studies have used a combination of: two-point linkage analysis, multipoint linkage analysis, haplotype analysis, and in some cases homozygosity mapping.

**Two-point/pairwise linkage:** considers each marker independently calculating the log of odds (LOD) score that each is linked to the disease locus. This method is particularly useful for identifying which of a number of highly polymorphic markers are closest to the mutated gene, and thus is very useful in restriction fragment length polymorphism (RFLP) and microsatellite-based mapping (see below).

**Multipoint linkage:** makes use of a genetic map, where the order of markers and the distance between them is specified. The probability that the disease locus lies at each point between these markers is then calculated sequentially.

**Haplotype Analysis:** involves analysing each pedigree in turn; considering the order of the markers along each chromosome and reconstructing the transmission of each allele across each generation and thus inferring the recombination events between markers to narrow down the disease interval. The explicit genotype at each marker which has the correct heritage can additionally be compared between unrelated samples, as if they are identical this may indicate they have the same origin (founder effect).

**Homozygosity mapping:** is applied to consanguineous families, and in considering the presumed autosomal recessive inheritance of the disease, identifies regions of the genome which exhibit this inheritance pattern.

A requirement of all these techniques is that uniquely identifiable markers are spaced throughout the genetic region of interest (be it the whole genome, chromosome, or smaller candidate region) which are then tested for proximity of the disease locus depending on which method is being applied. These markers have evolved over the last 15 years, which is reflected in the gene-finding approaches taken in autoinflammatory disease studies as described below.

### Restriction fragment length polymorphisms (RFLPs)

These are genetic markers where DNA is digested using restriction endonucleases before separation by gel electrophoresis and detection with a probe via Southern blot. A number of linkage studies in medium sized cohorts of families with Familial Mediterranean Fever (FMF) in the early 1990s were based on RFLP markers, identifying the short arm of chromosome 16 to harbour the afflicted gene for FMF in several populations [39-42] (and two studies based on microsatellites, below [5,6]), before the gene was subsequently identified [6,43].

### Microsatellites

The development of microsatellite markers allowed for the faster typing of a greater number of markers whilst consuming less DNA. Microsatellites are tandem repeats where the repeating unit consists of 2-6 bases and the repeat array covers between 10-1000bps. They can be amplified by PCR with primers in the unique flanking regions and detected by gel electrophoresis, or using fluorescent primers using an ABI prism genetic analyser allowing more markers to be detected at once. This type of genotyping has been at the core of mapping for a number of autoinflammatory diseases, including the four most common hereditary recurrent fevers.

McDermott et al. conducted multipoint and two-point linkage analysis using microsatellite markers in a large Irish-Scottish kindred and two additional Irish kindreds suffering from dominant periodic fevers, and identified an 8cM region on chromosome 12p13. Having observed a lower level of TNF receptor in the blood of patients they screened *TNFSFR1* as a candidate gene within that region [44]. This same region had also just been identified through multipoint linkage, two point linkage, and haplotype analysis in an Australian kindred of Scottish descent [7].

In 1999 pairwise linkage analysis was also central to the identification of the region harbouring the gene for Hyper IgD syndrome, conducted based on microsatellite markers in 13 families and then a candidate gene approach identifying the gene for mevalonate kinase (*MVK*) [8].

Two-point linkage, multipoint linkage and haplotype analysis on microsatellites were employed in the discovery of the gene *NLRP3* mutated in the Cryopyrinopathies. The fact that linkage in familial cold autoinflammatory syndrome (FCAS) and Muckle-Wells Syndrome (MWS) identified the same region, 1q44, highlighted that these were two diseases within a spectrum [9,10]. Neonatal-onset multisystem inflammatory disease (NOMID), also known as the chronic infantile neurological cutaneous and articular syndrome (CINCA), was later identified as the third condition to be caused by mutations in this gene after linkage studies triggered Feldman et al to consider *NLRP3* as a candidate gene [11].

### Single nucleotide polymorphisms (SNPs)

SNP arrays have further increased the number of markers which can be measured, though they are biallelic meaning heterozygosity is low, therefore a multipoint approach to linkage considering a greater number of markers at once has become critical. Genomic DNA is fragmented and hybridised to the array, which has DNA oligomers complementary to the sequence adjacent to the SNP. The alternate SNP alleles either affect the binding to either the oligomer and attached probe (Affymetrix arrays) or binding to a subsequent probe after single base pair (bp) extension (Illumina arrays). Consequently, this results in differential fluorescence of the probe which is detected when the array is scanned and allows for the base allele to be called.

*PSMB8* has been identified by three different groups looking at patients who were classified as having three differently named diseases. Agarwal et al studied patients with joint contractures, muscle atrophy, microcytic anaemia, and panniculitis-induced lipodystrophy syndrome (JMP) using homozygosity mapping and parametric multipoint linkage based on SNP genotypes from the Illumina HumanOmni1-Quad Beadchip [29]. Arima et al performed homozygosity mapping in five unrelated patients and three unaffected siblings of one of the patients with Nakajo-Nishimura Syndrome (NNS) using Affymetrix GeneChip Human Mapping 500K array set [30]; whilst Kitamura et al used homozygosity mapping and multipoint linkage to study Japanese autoinflammatory syndrome with lipodystrophy (JASL) patients and unaffected siblings from 2 consanguineous families genotyped on the Illumina Human 610 Quad combined with exome sequencing [31]. They found that both NNS and JASL was caused by the Gly201Val substitution (though Kitamura et al. refer to this as Gly197Val based on a different transcript of the gene) suggesting a founder effect. Meanwhile, Liu et al used a combination of homozygosity mapping with SNP genotypes from Affymetrix GeneChip Human Mapping 250K SNP Array, candidate gene sequencing and in one patient performed exome sequencing to identify *PSMB8* in Chronic Atypical Neutrophilic Dermatosis with Lipodystrophy and Elevated Temperature Syndrome (CANDLE) patients. This is a further example of how mapping can support whole exome sequencing [32].

SNP arrays have also enabled genome-wide association studies (GWAS) in polygenic diseases. Association studies employ a case-control experimental design, where DNA markers of a sample of unrelated individuals are measured to search for correlations with phenotype. For each marker the analysis is a simple 2x2 χ^2^ test. GWAS studies use high through-put platforms such as SNP arrays to detect if any of them are statistically associated with the disease. However, as each marker counts as an additional independent test, compensations must be made for this multiple testing and to surmount this threshold the number of cases and controls needs to be very large. Furthermore, the analysis may be perturbed by underlying population structures and ethnicities due to differences in allele frequencies. This must be carefully controlled or factored into the analysis. Once an association has been identified then there are rounds of further tests to confirm the putative association. Many of these studies use a course map of markers and then try to replicate the result in a further sample with more markers in the region. A number of GWAS have been undertaken in both Behçet’s disease (reviewed in [45]) and in Crohn’s disease [46].

### Deep sequencing based techniques - whole genome, whole exome and targeted capture sequencing

Whole genome sequencing takes the total genomic DNA from an individual and after preparing a DNA 'library’, sequencing the entire genome. In targeted capture, including whole exome sequencing, only the genomic regions of interest (e.g. all exons), are 'captured’ from the DNA library. This is done by hybridising the library to complementary DNA or RNA oligos and washing the unbound DNA away before eluting the bound DNA for sequencing. Exome capture kits can be bought 'off the shelf’; three of the biggest retailers of exome capture kits are Nimblegen, Agilent and Illumina, and the exact constitution of the exome depends on the proprietary composition of their capture oligos. Sequencing is then performed on one of the high throughput next generation sequencing (NGS) platforms – including Illumina’s Solexa sequencing by synthesis technologies, SOLiD sequencing by ligation, 454 pyrosequencing and Ion Torrent semiconductor technology.

Following sequencing are several bioinformatic processes including: aligning sequencing reads to the reference genome; variant calling which identifies deviations from the reference sequence; and annotation of variants. Annotation is the process of marking any variants found with information such as nearest gene, predicted consequences at protein level, and frequency in variant databases such as 1000 Genomes Project and SNP database (dbSNP) to help to eliminate common polymorphisms (occurring in >1 % of the healthy population). Based on these factors, certain variants may be selected as 'candidates’ for follow-up study with further insight into how the gene functions and whether the suspected variant is likely to interfere with this. Bioinformatic programs which may help with predicting consequences at protein level include PolyPhen [47] and MutationTaster [48].

For whole exome and whole genome sequencing this process can identify thousands of non-pathogenic variants per person. Sequencing multiple unrelated individuals is one solution to filter out non-pathogenic variants. Onoufriadis et al sequenced the exomes of 5 unrelated Europeans with generalised pustular psoriasis. In two of them they found the same homozygous mutation and a third was found to be a compound heterozygote in *IL36RN*[34], the gene now known to be associated with the autoinflammatory disease called deficiency of the interleukin 36 receptor antagonist (DITRA). Concurrent studies by another group used both homozygosity mapping combined with next generation sequencing, again leading to the identification of *IL36RN*, and emphasising the utility of combining these approaches for gene discovery [33].

Identifying the causal variant can be an enormous task if multiple unrelated patients are unavailable. For each variant the inheritance model can be considered, for example, if the disease is recessive is the variant homozygous? Runs of homozygous variants can also be identified effectively for homozygosity mapping [49]. The number of heterozygous variants from a sequenced exome which have the correct inheritance for autosomal dominant disease is more numerous than when considering homozygous or compound heterozygous variants for a recessive disease. Sequencing other family members can be informative to reconstruct the inheritance of variants. Using Agilent Sureselect 50Mb exome capture and SOLiD sequencing in a trio (an affected father and daughter and unaffected mother) enabled the identification of the *PLCG2* as being the gene mutated in Autoinflammatory PLCG2-associated antibody deficiency and immune dysregulation (APLAID) syndrome, an autosomal dominant disease [35].

Very rare SNP variants can be determined from deep sequencing, which may be an asset for genome-wide association studies of polygenic complex diseases [50]. This approach could be beneficial to polygenic autoinflammatory diseases such as periodic fever, aphthous ulceration, pharyngitis, and adenitis syndrome (PFAPA), Behçet’s disease, and has been applied in a small cohort in Crohn’s disease [51]. However, this is computationally complex and requires the typing of many samples from large patient cohorts to be sufficiently powered to detect genetic associations.

Increased sensitivity of the NGS technologies is also enabling detection of mutations in known genes present in a minority of cells, i.e. for the detection of somatic mosaicism. Approximately 50% of CINCA patients who have the classic features have no detected mutation in *NLRP3*, so-called "mutation negative CAPS". A significant proportion of these patients may have somatic mosaicism in their leucocytes causing their disease, but at a percentage of affected cells which is too low to be detected by conventional Sanger sequencing. Sensitivity of detection can be increased by the process of cloning into vectors, as these cells make up a small percentage (4.2-38.5%) of the total leucocyte population [52]. Alternatively, next generation sequencing technologies may detect low levels of somatic mosaicism and could become part of routine genetic screening in the future for CAPS, since the *NLRP3* and other related genes can be covered with sufficient read depth [Omoyinmi et al., manuscript submitted].

### Conclusion: clinical application and ethical considerations

We are in the midst of a revolution in diagnostic genetics in the field of autoinflammation. NGS technologies now allow rapid detection of novel genes causing autoinflammation in selected patients. In addition, techniques such as whole exome sequencing are increasingly being used in routine clinical practice as diagnostic tools to rapidly screen many genes, particularly for diseases where there is a phenotypic overlap and many possible genetic causes. In particular, carbohydrate deficient glycoprotein syndromes, and Charcot-Marie-Tooth disease are specific examples where whole exome sequencing allows rapid screening of many genes with greater efficiency and less cost than conventional Sanger sequencing [53]. Clearly, autoinflammatory diseases would lend themselves well to this sort of diagnostic approach since it is likely that in the future tens or even hundreds of genes will be implicated either singly or in combination as the cause of autoinflammation in certain patients. NGS technologies also bring new scientific challenges and ethical dilemmas. Our ability to discover new candidate genes now mandates that we develop robust functional experimental approaches to confirm that these genes are truly pathogenic, often the most challenging aspect of gene discovery in practice. At the same time, we are faced with new ethical considerations: the consequence of screening entire exomes and genomes means that unrelated/coincidental genetic disease may be identified. This raises complex ethical questions in relation to whether these findings should be communicated to the patient/family. This important issue in relation to "return of results" is reviewed elsewhere [54]. In the field of autoinflammation however, irrespective of these important ethical dilemmas, these new technologies have undoubtedly propelled us into an era of intense gene discovery and greater understanding of the immune system and its regulation that will rapidly be translated into better diagnostics and therapeutics for these patients.

## Competing interests

All authors declared that they have no competing interests.

## Authors’ contributions

AS wrote the manuscript. EO revised and added to the manuscript. PB revised the manuscript. All authors read and approved the final manuscript.

## References

[B1] McDermott MF Aksentijevich I Galon J McDermott EM Ogunkolade BW Centola M Mansfield E Gadina M Karenko L Pettersson T McCarthy J Frucht DM Aringer M Torosyan Y Teppo A-M Wilson M Karaarslan HM Wan Y Todd I Wood G Schlimgen R Kumarajeewa TR Cooper SM Vella JP Amos CI Mulley J Quane KA Molloy MG Ranki A Powell RJ **Germline mutations in the extracellular domains of the 55 kDa TNF receptor, TNFR1, define a family of dominantly inherited autoinflammatory syndromes** Cell 1999 97 133 144 10.1016/S0092-8674(00)80721-7 10199409

[B2] Kastner DL Aksentijevich I Goldbach-Mansky R Autoinflammatory disease reloaded: a clinical perspective Cell 2010 140 784 790 10.1016/j.cell.2010.03.002 20303869 PMC3541025

[B3] de Sarrauste Menthiere C Terrierre S Pugnere D Ruiz M Demaille J Touitou I INFEVERS: the registry for FMF and hereditary inflammatory disorders mutations Nucleic Acids Res 2003 31 282 285 10.1093/nar/gkg031 12520003 PMC165478

[B4] Dickie LJ Savic S Aziz A Sprakes M McDermott MF Periodic fever syndrome and autoinflammatory diseases F1000 med reports 2010 2 3 10.3410/M2-3 PMC2948378 20948856

[B5] French F.M.F.Consortium **Localization of the familial mediterranean fever gene to a 250-kb interval in non Ashkenazi Jewish founder haplotypes** Am J Hum Genet 1996 59 603 612 8751861 PMC1914916

[B6] French F.M.F.Consortium A candidate gene for familial Mediterranean fever Nat gene 1997 17 25 31 10.1038/ng0997-25 9288094

[B7] Mulley J Saar K Hewitt G Ruschendorf F Phillips H Colley A Sillence D Reis A Wilson M Gene localization for an autosomal dominant familial periodic fever to 12p13 Am J Human Gene 1998 62 884 889 10.1086/301793 PMC1377033 9529351

[B8] Drenth JPH Cuisset L Grateau G Vasseur C van de Velde-Visser SD de Jong JGN Beckmann JS van der Meer JWM Delpech M Mutations in the gene encoding mevalonate kinase cause hyper-IgD and periodic fever syndrome Nature genetics 1999 22 178 181 10.1038/9696 10369262

[B9] Cuisset L Drenth JPH Berthelot JM Meyrier A Vaudour G Watts RA Scott DGI Nicholls A Pavek S Vasseur C Beckmann JS Delpech M Grateau G Genetic Linkage of the Muckle-Wells Syndrome to Chromosome 1q44 Am J Hum Genet 1999 65 1054 1059 10.1086/302589 10486324 PMC1288238

[B10] Hoffman HM Wright FA Broide DH Wanderer AA Kolodner RD Identification of a Locus on Chromosome 1q44 for Familial Cold Urticaria Am J Hum Genet 2000 66 1693 1698 10.1086/302874 10741953 PMC1378006

[B11] Feldmann J Prieur AM Quartier P Berquin P Certain S Cortis E Teillac-Hamel D Fischer A Basile GS Chronic infantile neurological cutaneous and articular syndrome is caused by mutations in CIAS1, a gene highly expressed in polymorphonuclear cells and chondrocytes Am J Human Gene 2002 71 198 203 10.1086/341357 PMC384980 12032915

[B12] Tromp G Kuivaniemi H Raphael S Ala-Kokko L Christiano A Considine E Dhulipala R Hyland J Jokinen A Kivirikko S **Genetic linkage of familial granulomatous inflammatory arthritis, skin rash, and uveitis to chromosome 16** Am J Hum Genet 1996 59 1097 8900239 PMC1914842

[B13] Miceli-Richard C Lesage S Rybojad M Prieur AM Manouvrier-Hanu S Häfner R Chamaillard M Zouali H Thomas G Hugot JP CARD15 mutations in Blau syndrome Nat Genet 2001 29 19 20 10.1038/ng720 11528384

[B14] Ueki Y Tiziani V Santanna C Fukai N Maulik C Garfinkle J Ninomiya C DoAmaral C Peters H Habal M Rhee-Morris L Doss JB Kreiborg S Olsen BR Reichenberger E Mutations in the gene encoding c-Abl-binding protein SH3BP2 cause cherubism Nat Genet 2001 28 125 126 10.1038/88832 11381256

[B15] Tiziani V Reichenberger E Buzzo CL Niazi S Fukai N Stiller M Peters H Salzano FM Amaral CM R d Olsen BR **The gene for Cherubism maps to chromosome 4p16** Am J Hum Genet 1999 65 158 166 10.1086/302456 10364528 PMC1378086

[B16] Mangion J Rahman N Edkins S Barfoot R Nguyen T Sigurdsson A Townend JV Fitzpatrick DR Flanagan AM Stratton MR **The gene for Cherubism maps to chromosome 4p16.3** Am J Hum Genet 1999 65 151 157 10.1086/302454 10364527 PMC1378085

[B17] Wise CA Gillum JD Seidman CE Lindor NM Veile R Bashiardes S Lovett M Mutations in CD2BP1 disrupt binding to PTP PEST and are responsible for PAPA syndrome, an autoinflammatory disorder Hum Mol Genet 2002 11 961 969 10.1093/hmg/11.8.961 11971877

[B18] Wise CA Bennett LB Pascual V Gillum JD Bowcock AM Localization of a gene for familial recurrent arthritis Arthritis Rheum 2000 43 2041 2045 10.1002/1529-0131(200009)43:9<2041::AID-ANR15>3.0.CO;2-G 11014354

[B19] Yeon HB Lindor NM Seidman JG Seidman CE Pyogenic Arthritis, Pyoderma Gangrenosum, and Acne Syndrome Maps to Chromosome 15q Am J Human Gene 2000 66 1443 1448 10.1086/302866 PMC1288212 10729114

[B20] Kanazawa N Okafuji I Kambe N Nishikomori R Nakata-Hizume M Nagai S Fuji A Yuasa T Manki A Sakurai Y Nakajima M Kobayashi H Fujiwara I Tsutsumi H Utani A Nishigori C Heike T Nakahata T Miyachi Y Early-onset sarcoidosis and CARD15 mutations with constitutive nuclear factor-kappa B activation: common genetic etiology with Blau syndrome Blood 2005 105 1195 1197 15459013 10.1182/blood-2004-07-2972

[B21] Kanazawa N Matsushima S Kambe N Tachibana T Nagai S Miyachi Y Presence of a sporadic case of systemic granulomatosis syndrome with a CARD15 mutation J Invest Dermatol 2004 122 851 852 10.1111/j.0022-202X.2004.22341.x 15086578

[B22] Ferguson PJ Chen S Tayeh MK Ochoa L Leal SM Pelet A Munnich A Lyonnet S Majeed HA El-Shanti H Homozygous mutations in LPIN2 are responsible for the syndrome of chronic recurrent multifocal osteomyelitis and congenital dyserythropoietic anaemia (Majeed syndrome) J Med Genet 2005 42 551 557 10.1136/jmg.2005.030759 15994876 PMC1736104

[B23] Moglabey YB Kircheisen R Seoud M El Mogharbel N Van den Veyver I Slim R Genetic Mapping of a Maternal Locus Responsible for Familial Hydatidiform Moles Hum Mol Genet 1999 8 667 10.1093/hmg/8.4.667 10072436

[B24] Murdoch S Djuric U Mazhar B Seoud M Khan R Kuick R Bagga R Kircheisen R Ao A Ratti B **Mutations in NALP7 cause recurrent hydatidiform moles and reproductive wastage in humans** Nat Genet 2006 38 300 302 10.1038/ng1740 16462743

[B25] Jeru I Duquesnoy P Fernandes-Alnemri T Cochet E Yu JW Lackmy-Port-Lis M Grimprel E Landman-Parker J Hentgen V Marlin S McElreavey K Sarkisian T Grateau G Alnemri ES Amselem S **Mutations in NALP12 cause hereditary periodic fever syndromes** Proc Natl Acad Sci 2008 105 1614 1619 10.1073/pnas.0708616105 18230725 PMC2234193

[B26] Aksentijevich I Masters SL Ferguson PJ Dancey P Frenkel J van Royen-Kerkhoff A Laxer R Tedgard U Cowen EW Pham TH Booty M Estes JD Sandler NG Plass N Stone DL Turner ML Hill S Butman JA Schneider R Babyn P El-Shanti HI Pope E Barron K Bing X Laurence A Lee CC Chapelle D Clarke GI Ohson K Nicholson M **An autoinflammatory disease with deficiency of the interleukin-1-receptor antagonist** N Engl J Med 2009 360 2426 2437 10.1056/NEJMoa0807865 19494218 PMC2876877

[B27] Glocker EO Kotlarz D Boztug K Gertz EM Schaffer AA Noyan F Perro M Diestelhorst J Allroth A Murugan D Hatscher N Pfeifer D Sykora KW Sauer M Kreipe H Lacher M Nustede R Woellner C Baumann U Salzer U Koletzko S Shah N Segal AW Sauerbrey A Buderus S Snapper SB Grimbacher B Klein C **Inflammatory Bowel Disease and Mutations Affecting the Interleukin-10 Receptor** N Engl J Med 2009 361 2033 2045 10.1056/NEJMoa0907206 19890111 PMC2787406

[B28] Glocker EO Frede N Perro M Sebire N Elawad M Shah N Grimbacher B Infant colitis it's in the genes The Lancet 2009 376 1272 10.1016/S0140-6736(10)61008-2 20934598

[B29] Agarwal AK Xing C DeMartino GN Mizrachi D Hernandez MD Sousa AB Martinez De Villarreal L Dos Santos HG Garg A **PSMB8 encoding the B5i proteasome subunit is mutated in joint contractures, muscle atrophy, microcytic anemia, and panniculitis-induced lipodystrophy syndrome** Am J Hum Genet 2010 87 866 872 10.1016/j.ajhg.2010.10.031 21129723 PMC2997366

[B30] Arima K Kinoshita A Mishima H Kanazawa N Kaneko T Mizushima T Ichinose K Nakamura H Tsujino A Kawakami A Matsunaka M Kasagi S Kawano S Kumagai S Ohmura K Mimori T Hirano M Ueno S Tanaka K Tanaka M Toyoshima I Sugino H Yamakawa A Tanaka K Niikawa N Furukawa F Murata S Eguchi K Ida H Yoshiura K **Proteasome assembly defect due to a proteasome subunit beta type 8 (PSMB8) mutation causes the autoinflammatory disorder, Nakajo-Nishimura syndrome** Proc Natl Acad Sci 2011 108 14914 14919 10.1073/pnas.1106015108 21852578 PMC3169106

[B31] Kitamura A Maekawa Y Uehara H Izumi K Kawachi I Nishizawa M Toyoshima Y Takahashi H Standley DM Tanaka K Hamazaki J Murata S Obara K Toyoshima I Yasutomo K **A mutation in the immunoproteasome subunit PSMB8 causes autoinflammation and lipodystrophy in humans** J Clin Invest 2011 121 4150 4160 10.1172/JCI58414 21881205 PMC3195477

[B32] Liu Y Ramot Y Torrelo A Paller AS Si N Babay S Kim PW Sheikh A Lee CCR Chen Y Vera A Zhang X Goldbach-Mansky R Zlotogorski A **Mutations in PSMB8 cause CANDLE syndrome with evidence of genetic and phenotypic heterogeneity** Arthritis Rheum 2011 64 895 907 21953331 10.1002/art.33368 PMC3278554

[B33] Marrakchi S Guigue P Renshaw BR Puel A Pei XY Fraitag S Zribi J Bal E Cluzeau C Chrabieh M Towne JE Douangpanya J Pons C Mansour S Serre V Makni H Mahfoudh N Fakhfakh F Bodemer C Feingold J Hadj-Rabia S Favre M Genin E Sahbatou M Munnich A Casanova JL Sims JE Turki H Bachelez H Smahi A **Interleukin-36 Receptor Antagonist Deficiency and Generalized Pustular Psoriasis** N Engl J Med 2011 365 620 628 10.1056/NEJMoa1013068 21848462

[B34] Onoufriadis A Simpson M Pink A Di-áMeglio P Smith C Pullabhatla V Knight J Spain S Nestle F Burden A Capon F Trembath R Barker J **Mutations in IL36RN/IL1F5 Are Associated with the Severe Episodic Inflammatory Skin Disease Known as Generalized Pustular Psoriasis** Am J Hum Genet 2011 89 432 437 10.1016/j.ajhg.2011.07.022 21839423 PMC3169817

[B35] Zhou Q Lee GS Brady J Datta S Katan M Sheikh A Martins M Bunney T Santich B Moir S Kuhns D Priel D Ombrello A Stone D Ombrello MJ Khan J Milner JD Kastner D Aksentijevich I **A hypermorphic missense mutation in PLCG2, encoding phospholipase C gamma 2, causes a dominantly inherited autoinflammatory disease with immunodeficiency** Am J Hum Genet 2012 91 713 720 10.1016/j.ajhg.2012.08.006 23000145 PMC3484656

[B36] Boisson B Laplantine E Prando C Giliani S Israelsson E Xu Z Abhyankar A Israel L Trevejo-Nunez G Bogunovic D **Immunodeficiency, autoinflammation and amylopectinosis in humans with inherited HOIL-1 and LUBAC deficiency** Nat Immunol 2012 13 1178 1186 10.1038/ni.2457 23104095 PMC3514453

[B37] Fuchs-Telem D Sarig O Van-áSteensel M Isakov O Israeli S Nousbeck J Richard K Winnepenninckx V Vernooij M Shomron N Uitto J Fleckman P Richard G Sprecher E **Familial Pityriasis Rubra Pilaris is caused by mutations in CARD14** Am J Hum Genet 2012 91 163 170 10.1016/j.ajhg.2012.05.010 22703878 PMC3397268

[B38] Jordan CT Cao L Roberson E Pierson K Yang CF Joyce C Ryan C Duan S Helms C Liu Y Chen Y McBride A Hwu WL Wu JY Chen YT Menter A Goldbach-Mansky R Lowes M Bowcock A **PSORS2 is due to mutations in CARD14** Am J Hum Genet 2012 90 784 795 10.1016/j.ajhg.2012.03.012 22521418 PMC3376640

[B39] Pras E Aksentijevich I Gruberg L Balow JE Prosen L Dean M Steinberg AD Pras M Kastner DL **Mapping of a gene causing Familial Mediterranean Fever to the short arm of chromosome 16** N Engl J Med 1992 326 1509 1513 10.1056/NEJM199206043262301 1579134

[B40] Pras E Aksentijevich I Levy E Gruberg L Prosen L Dean M Pras M Kastner D The gene causing familial mediterranean fever maps to the short arm of chromosome 16 in Druze and Moslem Arab families Hum Genet 1994 94 576 577 7959700 10.1007/BF00211032

[B41] Gruberg L Aksentijevich I Pras E Kastner DL Pras M Mapping of the familial Mediterranean fever gene to chromosome 16 Am J Reprod Immunol 1992 28 241 10.1111/j.1600-0897.1992.tb00803.x 1285890

[B42] Shohat M Bu X Shohat T Fischel-Ghodsian N Magal N Nakamura Y Schwabe AD Schlezinger M Danon Y Rotter JI **The gene for familial Mediterranean fever in both Armenians and non-Ashkenazi Jews is linked to the alpha-globin complex on 16p: evidence for locus homogeneity** Am J Hum Genet 1992 51 1349 1463015 PMC1682901

[B43] The International F.M.F.Consortium Ancient missense mutations in a new member of the RoRet gene family are likely to cause familial Mediterranean fever Cell 1997 90 797 807 10.1016/S0092-8674(00)80539-5 9288758

[B44] McDermott MF Ogunkolade BW McDermott EM Jones LC Wan Y Quane KA McCarthy J Phelan M Molloy MG Powell RJ Amos CI Hitman GA **Linkage of Familial Hibernian Fever to Chromosome 12p13** Am J Hum Genet 1998 62 1446 1451 10.1086/301886 9585614 PMC1377165

[B45] Gul A Genome-wide association studies in Behcet's disease: expectations and promises Clin Exp Rheumatol 2011 29 S3 S5 21968232

[B46] Lee JC Parkes M Genome-wide association studies and Crohns disease Brief Funct Genomics 2011 10 71 76 10.1093/bfgp/elr009 21436303

[B47] Adzhubei IA Schmidt S Peshkin L Ramensky VE Gerasimova A Bork P Kondrashov AS Sunyaev SR A method and server for predicting damaging missense mutations Nat Methods 2010 7 248 249 10.1038/nmeth0410-248 20354512 PMC2855889

[B48] Schwarz JM Rodelsperger C Schuelke M Seelow D MutationTaster evaluates disease-causing potential of sequence alterations Nat Methods 2010 7 575 576 10.1038/nmeth0810-575 20676075

[B49] Su SY Kasberger J Baranzini S Byerley W Liao W Oksenberg J Sherr E Jorgenson E Detection of identity by descent using next-generation whole genome sequencing data BMC Bioinformatics 2012 13 121 10.1186/1471-2105-13-121 22672699 PMC3403908

[B50] Casals F Idaghdour Y Hussin J Awadalla P Next-generation sequencing approaches for genetic mapping of complex diseases J Neuroimmunol 2012 248 10 22 10.1016/j.jneuroim.2011.12.017 22285396

[B51] Ellinghaus D Zhang H Zeissig S Lipinski S Till A Jiang T Stade B Bromberg Y Ellinghaus E Keller A Rivas MA Skieceviciene J Doncheva NT Liu X Liu Q Jiang F Forster M Mayr G Albrecht M Hasler R Boehm BO Goodall J Berzuini CR Lee J Andersen V Vogel U Kupcinskas L Kayser M Krawczak M Nikolaus S **Association between variants of PRDM1 and NDP52 and Crohns disease, based on exome sequencing and functional studies** Gastroenterology 2013 145 339 347 10.1053/j.gastro.2013.04.040 23624108 PMC3753067

[B52] Tanaka N Izawa K Saito MK Sakuma M Oshima K Ohara O Nishikomori R Morimoto T Kambe N Goldbach-Mansky R **High incidence of NLRP3 somatic mosaicism in patients with chronic infantile neurologic, cutaneous, articular syndrome: results of an International Multicenter Collaborative Study** Arthritis Rheum 2011 63 3625 3632 10.1002/art.30512 21702021 PMC3498501

[B53] Ku C-S Cooper DN Polychronakos C Naidoo N Wu M Soong R Exome sequencing: dual role as a discovery and diagnostic tool Ann Neurol 2012 71 5 14 10.1002/ana.22647 22275248

[B54] Evans JP Rothschild BB Return of results: not that complicated? Genet Med 2012 14 358 360 10.1038/gim.2012.8 22481183

